# ‘It doesn't do the care for you': a qualitative study of health care professionals' perceptions of the benefits and harms of integrated care pathways for end of life care

**DOI:** 10.1136/bmjopen-2015-008242

**Published:** 2015-09-14

**Authors:** Katherine E Sleeman, Jonathan Koffman, Katherine Bristowe, Caroline Rumble, Rachel Burman, Sara Leonard, Jo Noble, Odette Dampier, William Bernal, Myfanwy Morgan, Philip Hopkins, Wendy Prentice, Irene J Higginson

**Affiliations:** 1 Department of Palliative Care Policy & Rehabilitation, King's College London, Cicely Saunders Institute, London, UK; 2 Intensive Care Unit, King's College Hospital NHS Foundation Trust, London, UK; 3 Division of Health and Social Care Research, King's College London, London, UK

**Keywords:** GENERAL MEDICINE (see Internal Medicine)

## Abstract

**Objectives:**

To understand healthcare professionals’ perceptions of the benefits and potential harms of integrated care pathways for end-of-life care, to inform the development of future interventions that aim to improve care of the dying.

**Design:**

Qualitative interview study with maximum variation sampling and thematic analysis.

**Participants:**

25 healthcare professionals, including doctors, nurses and allied health professionals, interviewed in 2009.

**Setting:**

A 950-bed South London teaching hospital.

**Results:**

4 main themes emerged, each including 2 subthemes. Participants were divided between (1) those who described mainly the benefits of integrated care pathways, and (2) those who talked about potential harms. Benefits focused on processes of care, for example, clearer, consistent and comprehensive actions. The recipients of these benefits were staff members themselves, particularly juniors. For others, this perceived clarity was interpreted as of potential harm to patients, where over-reliance on paperwork lead to prescriptive, less thoughtful care, and an absolution from decision-making. Independent of their effects on patient care, integrated care pathways for dying had (3) a symbolic value: they legitimised death as a potential outcome and were used as a signal that the focus of care had changed. However, (4) a weak infrastructure, including scanty education and training in end-of-life care and a poor evidence base, that appeared to undermine the foundations on which the Liverpool Care Pathway was built.

**Conclusions:**

The potential harms of integrated care pathways for the dying identified in this study were reminiscent of criticisms subsequently published by the Neuberger review. These data highlight: (1) the importance of collecting, reporting and using qualitative data when developing and evaluating complex interventions; (2) that comprehensive education and training in palliative care is critical for the success of any new intervention; (3) the need for future interventions to be grounded in patient-centred outcomes, not just processes of care.

**Strengths and limitations of this study**

- Qualitative research has an important role in shaping complex interventions to ensure that these are appropriate, acceptable and feasible in the chosen setting, but such approaches can be undervalued. This in-depth qualitative study examines healthcare professionals’ perceptions of the benefits and harms of integrated care pathways for end-of-life care in order to inform the development of interventions to improve care for the dying.
- We interviewed healthcare professionals from different grades of medical, surgical and allied specialties, and have developed detailed insights into the factors associated with successful implementation of integrated care pathways for end-of-life care.
- By using data collected in 2009, we are able to understand the views of healthcare professionals in the period before the media controversy surrounding the use of the Liverpool Care Pathway and Neuberger review.
- We interviewed a disproportionately large number of staff members from the intensive care unit, and participants were from a single tertiary referral centre, which may not be representative of the wider clinical setting.

## Introduction

Qualitative research, including exploration of patient, carer and healthcare professional perspectives, has an important role in shaping complex interventions to ensure that these are appropriate, acceptable and feasible. In the context of controlled trials, qualitative research can be used to understand the complexity of interventions and the context in which these are tested.^1^ However, qualitative research can be poorly integrated with other methods of evaluation, and may be undervalued.^2^

The Liverpool Care Pathway for the Dying Patient (LCP), an integrated care pathway for end-of-life care, was developed in England in the late 1990s.^3^ It aimed to distil the most important elements of good end-of-life care from the hospice setting, and transform these into a framework to guide and improve care in hospital, care home and community settings. The LCP provides prompts and guidance, within a structured single record, to promote the delivery of good care to people thought to be dying within hours or days, and was developed for use by doctors, nurses and allied health professionals inexpert in palliative care. It rapidly became suggested as a model of good practice by the UK Department of Health, and it formed an integral part of the National End of Life Care Programme.^4^ The LCP (or modified versions of it) was subsequently introduced in the USA, Australia, China and Europe.^5^

The aim of any integrated care pathway is to improve patient outcomes by promoting consistency and streamlining the processes of care.^6^ Although there was evidence that the LCP improved processes of care, for example, anticipatory prescribing of drugs for symptom control, prospective evidence of its benefits to patient outcomes, for example, improvement in symptoms, was lacking.^7–9^ In 2013, following intense media scrutiny in the British press of its potential harms, an independent review led by Baroness Neuberger identified numerous examples of poor care associated with the LCP, including poor communication, patchy senior decision-making and accounts of patients who appeared to have been oversedated or denied food and drink. The panel concluded that in the absence of reliable evidence of the pathway's benefits,^8^ ^9^ its use could no longer be justified.^10^

The extent to which healthcare professionals were aware of and in agreement with the potential harms exposed by the media and Neuberger review is unclear. Healthcare professionals’ views around the LCP were studied prior to the Neuberger review, but these studies cited mainly positive attitudes towards the impact of the LCP on the processes of care, for example, improvement in communication, continuity, documentation and as an educational tool.^11–14^ None of these cited harms similar to those reported in the Neuberger review. However, these studies were limited in terms of the population included.

Understanding the a priori reservations of healthcare professionals regarding potential harms of integrated care pathways for end-of-life care would help inform the implementation of the LCP outside the UK where it is still being used,^15^ and the development of any future interventions to improve care of the dying. We analysed data collected in 2009 as part of a mixed methods study to develop and implement a tool to improve palliative and end-of-life care in intensive care units (ICUs),^16^ a setting where end-of-life decision-making is complex and multifactorial. The original study collected data on the perceptions of healthcare professionals towards integrated care pathways for end-of-life care, including the LCP. The data were collected long before the issues were raised strongly in the British press and 4 years before the Neuberger review reported. One of the aims of the original study was to explore the views expressed by professionals about the potential benefits and harms of care pathways at the end of life. The findings are presented here, and compared with those issues subsequently identified by the Neuberger review.

## Methods

### Design

This was a qualitative analysis of interviews with healthcare professionals. The data were collected as part of a study which followed guidance for the development and evaluation of complex interventions from the Medical Research Council (the UK Government agency responsible for coordinating and funding medical research),^17^ and the Methods of Researching End-of-Life Care (MORECare) statement of good practice^18^ to develop and assess a tool to improve palliative and end-of-life care in the ICU. The original study and the results are published elsewhere.^16^ The study received full hospital research and development approval.

### Setting

The setting was two adult ICUs in a 950-bed South London teaching hospital, serving an area characterised by social deprivation and with culturally and ethnically heterogeneous populations. At the time of the study, the LCP had been implemented across much of the hospital, but was not used routinely in the intensive care setting.

### Participants

Maximum variation sampling was used to select potential staff participants to gain perspectives from a broad range of healthcare professionals by taking into account age, gender, profession and experience, and included both ICU and other hospital staff. Staff were identified through discussions with key staff members, and approached by letter or email. Written informed consent was gained from each participant prior to the interview. Twenty-five participants were interviewed: 13 nurses (junior to senior), 6 ICU doctors (junior to senior), 1 transplant coordinator, 2 social workers, 2 senior physicians and 1 senior surgeon. Three participants had extensive palliative care experience. All participants had some familiarity with integrated care pathways for end-of-life care, including the LCP, though experience varied by clinical settings and their grade. Interviews were carried out in 2009.

### Data collection

An interview time convenient to the healthcare professional was arranged (outside of clinical duties). Interviews were conducted in a confidential setting away from the clinical workplace unless the participant preferred not to. Interviews lasted 30–60 min and were conducted face-to-face with one of two trained interviewers (Dr Cathy Shipman (MSc), a senior research fellow and an experienced qualitative researcher, with an interest in non-specialist provision of palliative care; CR, a clinical research associate trained in qualitative methods). No relationship had been established prior to the start of the study, and there were no non-participants present. Topic guides were developed from a literature review; initial observations and discussions with service users, and explored perceptions, recommendations and views on integrated care pathways for palliative and end-of-life care (including the LCP); processes of decision-making; and experiences of palliative and end-of-life care. Although the study was based in the ICU, questions were more generally focused on the use of integrated care pathways. Questions were open-ended, and were piloted and revised. No repeat interviews were carried out. All interviews were digitally recorded and transcribed verbatim. The data were anonymised and code numbers allocated to each case. Themes were fed back and data were discussed with the project advisory group and with participants.

### Analysis

We used thematic analysis to inductively identify patterns and themes within the data. This approach utilises five related steps of: familiarisation, coding, theme development, defining themes and reporting.^19^ All interview data were reviewed during the process of familiarisation, and all sections of the interviews relating to the experience of utilising integrated care pathways were extracted. Emergent themes were identified from the data, defined and reported through an iterative process of theme development.

The primary data coder was KES. Specialist software was not used. To address issues of analytical rigour and trustworthiness, a subset of transcripts were double-coded by KB. A reiterant process of discussing areas of agreement and disagreement took place between KES and KB to achieve consensus. Alternative interpretations were incorporated into the analysis. The analysis was further tested during discussions with colleagues, and meetings of the project advisory steering group. We also paid attention to non-confirmatory cases where emerging themes contradicted more common ideas. Quotations were chosen to illustrate the themes, and to include a range of study participants.

## Results

**Figure 1:**
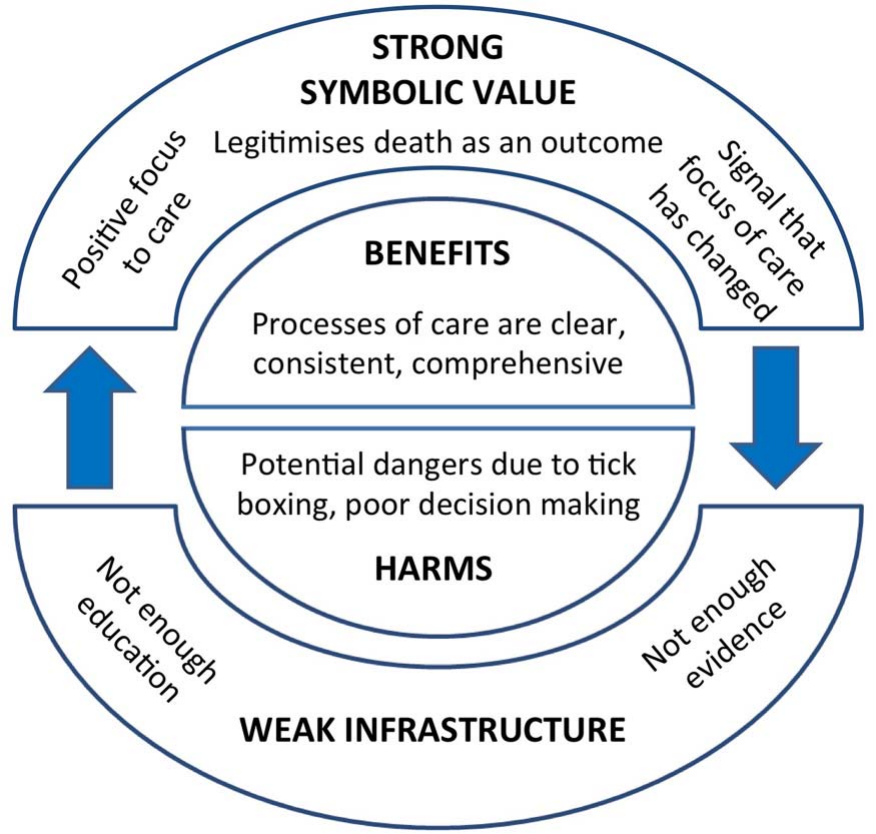
Model of healthcare professionals’ perceptions of the benefits and harms of integrated care pathways for end-of-life care.

Four themes were identified from the interview transcripts, each including two subthemes. Participants were divided between those who cited mainly benefits of integrated care pathways, and those who talked about potential harms. In addition, integrated care pathways for dying appeared to have a symbolic value, acting as a signal that the focus of care had changed. Underlying this were comments relating to the context and infrastructure within which care was provided (figure 1).

### The benefits of integrated care pathways for the dying

#### Processes of care

Many participants cited benefits of integrated care pathways for dying with respect to processes of care. The LCP provides a structured single record with prompts to guide care, and nursing staff, in particular, appeared to value the structure that integrated care pathways provide. There was frequent mention of improved clarity about the care which was to be provided.

I think perhaps it does mean that everybody has a clear picture as to what we are doing and not doing. (Senior nurse)

Integrated care pathways were felt to make care of the dying more consistent and comprehensive, and were felt to be particularly valuable in situations where continuity was compromised, for example, out of hours or when turnover of staff (medical or nursing) was high.

The biggest challenge I find as a nurse is not really knowing where you stand sometimes with treatment with, you know, perhaps the weekend…that's why I think the pathway is a good thing because it gives people guidance and gives us nurses something to follow. (Senior nurse)

An extreme view was that integrated care pathways for end-of-life care could provide a substitute for face-to-face handover between healthcare professionals changing shifts.

So having a form…can make sure that everybody involved can see where you are, what your aims are and what the plan actually is,…rather than having to, to discuss the plan for 10 minutes and tell the new person, you know…if there's a form then everybody can just see, sort of what we're doing. (Senior ICU doctor)

From many study participants there was a sense that integrated care pathways provide absolute clarity about processes of care. Much of the language used was of process and protocol, rather than uncertainty or grey areas.

so it's clear on every patient this is what we're going to do, this is the process. (Junior nurse)

#### Influence of healthcare provider experience

Several participants thought integrated care pathways for dying people were particularly beneficial for the more junior or inexperienced healthcare professionals. Again, this was particularly related to processes of care: care pathways provided clarity and structure to the care delivered.

But I think it provides clear guidelines, in my experience, for junior staff to follow and it is very clear and easy to follow and I think it provides a nice framework. (Junior doctor)

However, a minority of interviewees expressed a different view: that integrated care pathways may be particularly poorly used by inexperienced staff. For example, a consultant surgeon spoke about care pathways being used too rigidly by inexperienced colleagues.

I think pathways…give some kind of guideline which is helpful for people but often it is particularly young colleagues, it is something which makes them more inflexible…in the way that the most important thing if you work with pathways is to identify patients who don't fit in to the pathways because otherwise you make wrong decisions based on your pathways. (Senior surgeon)

### The potential harms of integrated care pathways for the dying

#### Patient outcomes

In this study, it was uncommon for staff members to talk about the impact of integrated care pathways on patient outcomes, and no participant spoke about integrated care pathways as improving the quality of patients’ deaths. Where outcomes were discussed, these related to the potential for harm. The words ‘dangerous’ and ‘danger’ were used. Integrated care pathways were not thought to be intrinsically bad, but were susceptible to poor use.

The distinction between processes of care and patient outcomes was highlighted clearly by one participant.

but it is documentation so it doesn't do the care for you,…and there's still an awful lot of thought and…work that you know needs to go into giving that care,…so it's not a tick box exercise,…and I think there's just a danger of that. (Senior nurse)

#### Tick-box care

Several study participants expressed concern that end-of-life care needs to be individualised, and that the structure of integrated care pathways, which in the case of the LCP included 4 and 12 hourly prompts, can inhibit the necessary flexibility required to provide good care to the dying. One participant spoke about integrated care pathways promoting tick-box care and inhibiting thoughtfulness.

Whatever care pathway there is, I'm always worried about people switching off their brains. Tick-boxing. Putting down on paper what they have to to fill in the paperwork. (Senior ICU doctor)

Another participant with extensive experience in palliative care spoke about the tension between providing holistic end-of-life care and following a pathway, and suggested that integrated care pathways may absolve healthcare professionals from clinical decision-making.

I think it's dangerous at the moment at times because that clinical decision-making doesn't happen, it isn't documented and in some instances the pathway, and that's not the intention of the pathway and the people who developed the pathway, but the presence of the pathway, the options of the pathway actually seems to absolve people from that. (Senior physician)

### The symbolic value of integrated care pathways

#### A signal

Several participants described integrated care pathways for end-of-life care in a way that suggested a symbolic value. Pathways were considered to be a useful signal, even before the paperwork was filled in, to herald the change in focus of care from active to palliative treatment. For example, the presence of the paperwork at the bedside was described as a non-verbal form of communication that the focus of care had changed.

When it does become a focus issue we very quickly get the paperwork out of the stationery cupboard and put it there [by the bed]. (Senior nurse)

#### A change in focus

The availability of integrated care pathways for end-of-life care was felt to have a value in legitimising death as an outcome in hospital, providing an acceptable alternative to aggressive medical care. One ICU consultant spoke about integrated care pathways providing a positive focus to the change in patient care by highlighting the care which will be provided, rather than aspects of care that are thought to be no longer appropriate.

There was a sense, however, that the use of integrated care pathways may promote a binary attitude towards the dying: integrated care pathways are either used or not used; therefore, patients are either dying or not dying. This perceived clarity regarding the change in focus of care may give some clinicians permission to distance themselves from the patient's care entirely. One senior physician talked about ‘switching off’ when the LCP is used.

I always joke about departments where, yes, the Liverpool Care Pathway is used in the department, do you know exactly what happens, by that stage you've switched off and you've handed the patient over, I think that's the honest truth. (Senior physician)

### Infrastructure

#### Education and training

Education and training in palliative care were commented only occasionally. A few study participants volunteered that they had received palliative care training, and for those who had, this had often focused more on how to use the LCP paperwork than generic palliative care skills. For one participant, the LCP itself appeared to act as an educational tool.

things like the Liverpool Care Pathway and things like that, I think they are a distillation of what I personally have been taught piecemeal over 10, 11 years now since graduation, and even before. (Senior ICU doctor)

#### Evidence

Only one study participant spoke about the evidence base for the LCP. This participant expressed concern about the lack of strong evidence of the benefits of the LCP, and the lack of awareness among other medical colleagues about the paucity of evidence.

There is no evidence. It's not a validated tool. (Senior physician)

The four main themes outlined above are illustrated in figure 1. The focus of this study was to examine healthcare professionals’ perceptions of benefits and/or harms of integrated care pathways for care of the dying, and individual participants were divided into those who cited mainly benefits, and those who cited harms. Central to understanding these experiences is the wider context in which integrated care pathways were implemented. An infrastructure inadequately supported by evidence and education may have paradoxically led to inflation of its symbolic value, and this in turn allowed shortcomings in evidence to be overlooked.

## Discussion

This study demonstrates that in the years preceding the Neuberger review, healthcare professionals were conscious of both benefits and harms of integrated care pathways for end-of-life care. The benefits related to streamlined processes of care, and were experienced by the healthcare professionals themselves. Potential harms related to applying the pathway inflexibly or without thinking, leading to poor clinical decision-making, and were reminiscent of criticisms subsequently published by the media and the Neuberger review.

It is notable that no participant in this study cited benefits regarding improved outcomes for patients. This does not mean that integrated care pathways do not have the potential to improve the quality of patients’ end-of-life care, but it does suggest that healthcare professionals using them may lose sight of the ultimate goal of care: a good death for the patient and improved outcomes in bereavement for their carers. The LCP audits, which measured success of implementation based on process measures and not patient-centred outcomes, may have reinforced this aspect.^20^

The LCP was not intended as a protocol, but as a guide.^21^ We found that it was often interpreted as a protocol, and moreover for many staff members, particularly the more junior clinicians, this aspect was particularly valued. This may be because training and education in palliative care had been fragmented and unsystematic, for example, focused on documentation or provided ‘piecemeal’ over years. It was the more senior clinicians who identified potential harms of integrated care pathways in this study, including relying on these too heavily as protocols. This may reflect their more extensive clinical experience, and overall responsibility for patient care. Integrated care pathways are not a substitute for skills, knowledge or expertise, but there may be a tendency for professionals, particularly those most junior, to interpret them as such. The importance of specialist palliative care team support and specialist training when implementing integrated care pathways for end-of-life care was highlighted in the first randomised trial of the LCP, published 6 months after the Neuberger review.^22^ ^23^

Although the LCP was intended simply as a guide to care, it fulfilled additional roles. An integrated care pathway for end-of-life care acted as a symbol to herald the change from curative to palliative treatment, to signal to others that the focus of care had changed and to legitimise that change. Indeed, for some clinicians, the perceived clarity of this switch in the focus of care appeared to allow them to distance themselves from the patients’ care entirely.

One of the main criticisms made by the Neuberger review was the lack of prospective testing of the LCP. It is interesting that only one healthcare professional in this study cited the importance of knowing the evidence base for such a pathway. Professionals may consider research evidence less important or relevant when people are dying, and this may be compounded by the historical paucity of research funding for palliative care.^24^ Patchy education and training in palliative care may have created a vacuum that allowed a tool for which there was no strong evidence to become accepted and valued. The strong symbolic value of the LCP may, in turn, have made it easier for professionals, as well as institutions and policymakers, to overlook the shortcomings in evidence (as illustrated in figure 1).

The strengths of this study are that a large number of people were interviewed, from different grades within several medical, surgical and allied specialties. By using data collected in 2009, we are able to understand the views of healthcare professionals in the period before the media controversy and Neuberger review.

This study has limitations. The original study focused on those dying in the ICU, where rapid changes in health status and prognostic uncertainty are common. The disproportionately large number of staff members from the ICU in this study may, therefore, not be representative of the wider clinical setting. Attitudes towards integrated care pathways for end-of-life care were just one part of the original study and the interview schedule.

This study has important implications for the future development of interventions to improve end-of-life care. First, it demonstrates the importance of collecting, reporting, and using qualitative data during the development of complex interventions.^25^ All interventions have benefits and harms, some of which may be obvious, others less so, especially when the intervention is complex.^26^ Collecting such data, including patient, carer and healthcare professional perspectives, during the early implementation of an integrated care pathway for end-of-life care would enable it to be refined and improved.

Second, the study emphasises the importance of investment in education and training in palliative care. The enthusiasm for what was perceived to be a protocol for end-of-life care indicates a need for improved understanding of how to care for the dying. Without these generic skills, it is unlikely that staff members would be able to use any such tools well or to recognise when these tools are being used poorly.

Third, the study identifies the importance of grounding the development of any future tools to improve care of the dying around patient outcomes, not just processes. Measuring processes of care is often more straightforward than measuring outcomes. However, reliance on process measures not only meant that it was impossible to demonstrate whether the LCP improved quality of care for patients and families,^10^ it may also have contributed to the staff who used it losing sight of the overall goals of care. Re-orientating healthcare professionals from processes to patient-centred outcomes is necessary to improve end-of-life care.

## References

[1] O'Cathain A, Thomas KJ, Drabble SJ What can qualitative research do for randomised controlled trials? A systematic mapping review. BMJ Open 2013;3:pii: e002889 10.1136/bmjopen-2013-002889 PMC3669723 23794542

[2] Lewin S, Glenton C, Oxman AD Use of qualitative methods alongside randomised controlled trials of complex healthcare interventions: methodological study. BMJ 2009;339:b3496 10.1136/bmj.b3496 19744976 PMC2741564

[3] Ellershaw J, Wilkinson S Care for the dying: a pathway to excellence. Ellershaw, Wilkinson, eds. Oxford, Oxford University Press, 2003.

[4] Department of Health End of life care strategy—promoting high quality care of all adults at the end of life. 2008.

[5] Phillips JL, Halcomb EJ, Davidson PM End-of-life care pathways in acute and hospice care: an integrative review. J Pain Symptom Manage 2011;41:940–55. 10.1016/j.jpainsymman.2010.07.020 21398083

[6] Campbell H, Hotchkiss R, Bradshaw N Integrated care pathways. BMJ 1998;316:133–7. 10.1136/bmj.316.7125.133 9462322 PMC2665398

[7] Currow DC, Abernethy AP Lessons from the Liverpool Care Pathway—evidence is key. Lancet 2014;383:192–3. 10.1016/S0140-6736(13)62039-5 24139707

[8] Chan RJ, Webster J End-of-life care pathways for improving outcomes in caring for the dying. Cochrane Database Syst Rev 2013;11:CD008006 10.1002/14651858.CD008006.pub3 24249255

[9] Parry R, Seymour J, Whittaker B Rapid evidence review: pathways focused on the dying phase in end of life care and their key components. Nottingham: University of Nottingham and the NHS End of Life Care Programme, 2013:5.

[10] Department of Health More care, less pathway: a review of the Liverpool Care Pathway. London: 2013.

[11] Paterson BC, Duncan R, Conway R Introduction of the Liverpool Care Pathway for end of life care to emergency medicine. Emerg Med J 2009;26:777–9. 10.1136/emj.2008.067249 19850797

[12] Gambles M, Stirzaker S, Jack BA The Liverpool Care Pathway in hospices: an exploratory study of doctor and nurse perceptions. Int J Palliat Nurs 2006;12:414–21. 10.12968/ijpn.2006.12.9.21869 17077800

[13] Jack BA, Gambles M, Murphy D Nurses’ perceptions of the Liverpool Care Pathway for the dying patient in the acute hospital setting. Int J Palliat Nurs 2003;9:375–81. 10.12968/ijpn.2003.9.9.11764 14593273

[14] Di Leo S, Beccaro M, Finelli S Expectations about and impact of the Liverpool Care Pathway for the dying patient in an Italian hospital. Palliat Med 2011;25:293–303. 10.1177/0269216310392436 21239466

[15] Raijmakers N, Dekkers A, Galesloot C Barriers and facilitators to implementation of the Liverpool Care Pathway in the Netherlands: a qualitative study. BMJ Support Palliat Care 2015;5:259–65. 10.1136/bmjspcare-2014-000684 25200707

[16] Higginson IJ, Koffman J, Hopkins P Development and evaluation of the feasibility and effects on staff, patients, and families of a new tool, the Psychosocial Assessment and Communication Evaluation (PACE), to improve communication and palliative care in intensive care and during clinical uncertainty. BMC Med 2013;11:213 10.1186/1741-7015-11-213 24083470 PMC3850793

[17] Craig P, Dieppe P, Macintyre S Developing and evaluating complex interventions: the new Medical Research Council guidance. BMJ 2008;337:a1655 10.1136/bmj.a1655 18824488 PMC2769032

[18] Higginson IJ, Evans CJ, Grande G Evaluating complex interventions in end of life care: the MORECare statement on good practice generated by a synthesis of transparent expert consultations and systematic reviews. BMC Med 2013;11:111 10.1186/1741-7015-11-111 23618406 PMC3635872

[19] Miles M, Huberman A Qualitative data analysis. London: Sage, 1994.

[20] National Care of the Dying Audit—Hospitals. https://www.rcplondon.ac.uk/sites/default/files/national_care_of_the_dying_audit_-_hospitals_exec_summary.pdf, Royal College of Physicians, 2012

[21] Murphy D The Liverpool Care Pathway provides clarity and focus; communication, care, and compassion come from you. Int J Palliat Nurs 2011;17:529 10.12968/ijpn.2011.17.11.529 22240629

[22] Costantini M, Romoli V, Leo SD, *et al*. Liverpool Care Pathway for patients with cancer in hospital: a cluster randomised trial. *Lancet* 2013. 10.1016/S0140-6736(13)61725-0 24139708

[23] Di Leo S, Romoli V, Higginson IJ ‘Less ticking the boxes, more providing support’: a qualitative study on health professionals’ concerns towards the Liverpool Care of the Dying Pathway. Palliat Med 2015;29:529–37. 10.1177/0269216315570408 25690601

[24] Sleeman KE, Gomes B, Higginson IJ Research into end-of-life cancer care—investment is needed. Lancet 2012;379:519 10.1016/S0140-6736(12)60230-X 22325658

[25] Campbell M, Fitzpatrick R, Haines A Framework for design and evaluation of complex interventions to improve health. BMJ 2000;321:694–6. 10.1136/bmj.321.7262.694 10987780 PMC1118564

[26] Currow DC, Higginson I Time for a prospective study to evaluate the Amber Care Bundle. BMJ Support Palliat Care 2013;3: 376–7. 10.1136/bmjspcare-2013-000608 24950514

